# An Immunological Review of SARS-CoV-2 Infection and Vaccine Serology: Innate and Adaptive Responses to mRNA, Adenovirus, Inactivated and Protein Subunit Vaccines

**DOI:** 10.3390/vaccines11010051

**Published:** 2022-12-26

**Authors:** Suhaila A. Al-Sheboul, Brent Brown, Yasemin Shboul, Ingo Fricke, Chinua Imarogbe, Karem H. Alzoubi

**Affiliations:** 1 Faculty of Applied Medical Sciences, Medical Laboratory Sciences, University of Science and Technology, Jordan; 2 Biochem123Education, London, UK; 3 Independent Researcher, Germany; 4 UKHSA, Rosalind Franklin Laboratory, UK; 5 College of Pharmacy, University of Sharjah, Sharjah, United Arab Emirates; 6 Faculty of Pharmacy, Jordan University of Science and Technology, Irbid, Jordan

**Keywords:** COVID-19 vaccines, Pfizer, BioNTech, Oxford–AstraZeneca, Sinopharm, Novavax, antibody response, T cell, B cell, neutralizing antibodies, adaptive immune response, immunology

## Abstract

The coronavirus disease 2019 (COVID-19) pandemic is caused by the severe acute respiratory syndrome coronavirus 2 (SARS-CoV-2) virus, which is defined by its positive-sense single-stranded RNA (ssRNA) structure. It is in the order Nidovirales, suborder Coronaviridae, genus Betacoronavirus, and sub-genus Sarbecovirus (lineage B), together with two bat-derived strains with a 96% genomic homology with other bat coronaviruses (BatCoVand RaTG13). Thus far, two Alphacoronavirus strains, HCoV-229E and HCoV-NL63, along with five Betacoronaviruses, HCoV-HKU1, HCoV-OC43, SARS-CoV, MERS-CoV, and SARS-CoV-2, have been recognized as human coronaviruses (HCoVs). SARS-CoV-2 has resulted in more than six million deaths worldwide since late 2019. The appearance of this novel virus is defined by its high and variable transmission rate (RT) and coexisting asymptomatic and symptomatic propagation within and across animal populations, which has a longer-lasting impact. Most current therapeutic methods aim to reduce the severity of COVID-19 hospitalization and virus symptoms, preventing the infection from progressing from acute to chronic in vulnerable populations. Now, pharmacological interventions including vaccines and others exist, with research ongoing. The only ethical approach to developing herd immunity is to develop and provide vaccines and therapeutics that can potentially improve on the innate and adaptive system responses at the same time. Therefore, several vaccines have been developed to provide acquired immunity to SARS-CoV-2 induced COVID-19-disease. The initial evaluations of the COVID-19 vaccines began in around 2020, followed by clinical trials carried out during the pandemic with ongoing population adverse effect monitoring by respective regulatory agencies. Therefore, durability and immunity provided by current vaccines requires further characterization with more extensive available data, as is presented in this paper. When utilized globally, these vaccines may create an unidentified pattern of antibody responses or memory B and T cell responses that need to be further researched, some of which can now be compared within laboratory and population studies here. Several COVID-19 vaccine immunogens have been presented in clinical trials to assess their safety and efficacy, inducing cellular antibody production through cellular B and T cell interactions that protect against infection. This response is defined by virus-specific antibodies (anti-N or anti-S antibodies), with B and T cell characterization undergoing extensive research. In this article, we review four types of contemporary COVID-19 vaccines, comparing their antibody profiles and cellular aspects involved in coronavirus immunology across several population studies.

## 1. Introduction

The current COVID-19 pandemic that began in 2019 is caused by a pathogenic novel SARS-CoV-2 virus that causes infection predominantly through respiratory transmission, with varying clinical presentation affected by co-morbidities and other unknown factors (e.g., bacterial mutation or coinfections). Immunological responses are variable and affected by cellular and molecular as well as variable genetic characteristics to be discussed in detail in our next paper, provisionally entitled “Innate and Adaptive Immune Biomolecular Mechanisms by Case Study within SARS-CoV-2Pathogenesis”. The SARS-CoV-2 virion is composed of four predominant protein structures: spike (S protein), nucleocapsid (N protein), envelope (E protein), and membrane (M protein) [1,2,3]. The genome size of SARS-CoV-2 is approximately 30 kilobases and is defined by open reading frames (ORFs) that encode 16 non-structural proteins (NSP) necessary for amino acid synthesis via viral attachment, penetration, uncoating, replication, assembly, and virion release [4]. SARS-CoV-2 infects numerous cells via respiratory pathways, initially using ACE2 as the predominant receptor for receptor-mediated entry [5]. However, other receptors are implicated in independently mediated cell signaling and entry, including a type II transmembrane protease (TMPRSS2), asialoglycoprotein receptor-1 (ASGR1), kringle-containing transmembrane protein 1 (KREMEN1), dipeptidyl peptidase 4 (DPP4), neuropilin (NRP1), and CD147 amongst others. These receptors are awaiting further research clarification, while others are currently being investigated [6,7,8,9]. Humans infected with COVID-19 display both asymptomatic and symptomatic acute to chronic pathologies, much like other coronaviruses (SARS-CoV-1 and MERS) and other seasonal respiratory viral pathogens that also include influenza and respiratory syncytial virus (RSV) that cause a range of symptoms during viral pathogenesis [10,11,12]. The rate of infection and mortality is defined by R0 (growth rate) and infection fatality rate (IFR). Current R0 estimates vary from 1.47 to 1.86, with IFR estimates ranging between 0.49 and 2.53. However, these values are affected by spike protein mutational variation, with an array of vaccines being re-developed alongside other modern therapeutics [13,14]. In March 2020, the World Health Organization officially announced COVID-19 to be a pandemic [1,2,3,15,16,17]. Therefore, vaccines represent the primary-infection-management prophylactic approach, providing protection by developing an acquired immunity against SARS-CoV-2 and reducing the disease burden on healthcare systems [18,19]. SARS-CoV-2 causes COVID-19 infection mainly via the S-protein, with which the virus enters cells via the angiotensin-converting enzyme 2 receptor (ACE2) [20]. Neutralizing antibodies (nAbs) are generated against specific epitopes of the S, N, M and E protein antigens during SARS-CoV-2 infection [21,22,23]. Vaccine preparation and research has focused on the S protein as the primary target for blocking receptor-mediated entry, although N protein therapeutics are being developed [24]. S proteins are structured into an S1 subunit and S2 subunit that exist in the viral envelope as homotrimers. The S1 subunit determines receptor recognition via a receptor-binding domain (RBD), whereas the S2 subunit is responsible for membrane fusion and entry by binding to the angiotensin-converting enzyme 2 (ACE2) that covers human respiratory epithelial cells, resulting in the enabling of the virus RNA attachment and intracellular replication that occurs during SARS-CoV-2 infection [25,26]. The S1 domain comprises a signal peptide (SP), RBD, and S2 fusion protein. The S2 domain encompasses various other proteins including N-terminal domain (NTD), RBD, and C-terminal domains (CTD1 and CTD2). The S2 domain has additional fusion peptides (FP) alongside two heptad repeat domains (HR1 and HR2), transmembrane domain, and a C-terminal domain with a furin cleavage site [27,28]. S protein fusion can also utilize TMPRSS2 and endosomal cysteine proteases thought to mediate SARS-CoV-2 entry depending on spike and ACE2 conformation [29,30]. Many COVID-19 vaccines have now been produced that elicit B cell evoked IgG anti-S protein antibodies that demonstrate chronic disease prevention [31].

As of 30 October 2022, there are 276 potential therapeutics and 332 therapeutics under development for COVID-19 prevention, according to current data from the Milken Institute (milkeninstitute.org). Among these, 10 vaccines began adult human clinical trials in different phases [26]. The research and development of cellular mechanisms and proteins involved in vaccine development have become priorities to improve the understanding of COVID-19 pathogenesis, which will lead to an expansion in the understanding of other pathologies. After clinical trials, it was found that nAbs and non-nAbs do evoke humoral and cellular immunity, which perform the essential roles required in protection from serious disease in the majority after at least one vaccine dose, depending on previously diagnosed clinical conditions [1,32,33].

Vaccine development utilizes various protein-modification platforms, which include inactivated vaccines combined with adjuvant material (e.g., polio), live attenuated vaccines (e.g., MMR), protein subunits (influenza), and virus-like particles (e.g., HPV) [34]. More recently, advances in other platforms have utilized newer technologies, including those of viral vectors (e.g., VZV and Ebola), DNA (nucleic acid) vaccines, and RNA vaccines [18,34,35]. According to the SARS-CoV-2 research and other comparator studies of HCoV, there is a high level of B cell IgG produced against the S and N proteins, indicating that prior chronic COVID-19 infection or vaccination may provide excellent protection against SARS-CoV-2 re-infection [36,37,38].

## 2. Methodology

A review of peer reviewed articles and reports that were published in electronic databases, such as PubMed, bioxRiv, and Google Scholar, was conducted between September 2020 and October 2022; articles were chosen using the following keywords: “COVID-19 Vaccine”, “immune responses after COVID-19”, “SARS-CoV-2”, “Coronavirus”, “Pfizer vaccine”, “Vaccine Immunity”, “Vaccine efficacy”, “Antibody responses”, “Memory B cells”, “Sinopharm vaccine”, “AstraZeneca vaccine”, “Vaccine immunogenicity”, “Pfizer vaccine immune response”, “Oxford-AstraZeneca”, “BioNTech/Pfizer”, “Sinopharm/BBIBP-CorRV,” “effectiveness”, “ChAdOx1,” “Vaxzevria,” “Covishield,” “BNT162b1,” and “BNT162b2”.

Articles were chosen and analyzed in accordance with the relevant topics.

### 2.1. Factors Involved in SARS-CoV-2 Serological Analysis

An immune response is defined in research immunological terms by quantifying the antibody type, cellular markers, and cytokines relevant to the antigen protein presented by the pathogen for immune system recognition, particularly in the case of the novel SARS-CoV-2 infection. Leukocytes or white blood cells, specifically B lymphocytes, produce the predominant antibody immunoglobulin G (IgG), which will be discussed here in the context of innate and adaptive immune responses [39]. Our next article will present further specific details regarding specific cellular markers documented within other immune cells, including monocytes, dendritic cells, and others. B cells have a multifaceted function in pathologies, including viral infection, cytokine release, antigen processing, presentation, and antibody secretion. Serum IgG is mainly elicited during natural infection or vaccine-induced immune responses and can be measured in real-world settings via enzyme-linked immunosorbent analysis (ELISA) and respective antibody sub-types (IgG, IgA, IgD, IgM, IgE). The currently available research profiles mainly B cells that produce IgG in response to SARS-CoV-2, which is the most readily available and stable protein used in such assays; this protein is then validated as specific against viral antigens such as S protein. However, reagents used to quantify anti-SARS-CoV-2 specificity undergo further validation for specificity and sensitivity. Therefore, other studies (*n* = 87) quantify comparable assay sensitivities of IgA, IgM, and IgG in ratios of 98.6%: 96.8%:96.8%, respectively, with specificities of 98.1%, 92.3%, and 99.8% [40]—guiding the current standardization of such assays across manufacturers [41]. However, in other respiratory pathogens, for example influenza, the antibody response in the upper respiratory tract is dominated by IgA, which has a serum and secretory component. IgA is also produced in the mucosa-associated lymphoid tissue (MALT), primarily in the lamina propria, and then actively transported to mucosal surfaces through interactions with the polymeric immunoglobulin receptor [42]. For SARS-CoV-2, it is suggested by La Salle et al. that other antibody subtypes evoked in chronic COVID-19 patients play a key role, with expressions of high levels of IgM, IgG1, IgA1, IgG2, and IgG3 antibodies throughout infection that require further research. It is currently thought that IgG1 and IgG3 correlate with SARS-CoV-2 severity [43]. IgG2 could be more important in bacterial responses to capsular polysaccharide antigens [44]. Limited data currently exist to accurately reveal why SARS-CoV-2 exhibits such a novel antibody profile in chronic disease regarding IgG1/IgA1 responses [45]. However, earlier this year, Kober et al. performed an analysis indicating that IgG3 and IgM could be responsible for 80% of the overall neutralization of SARS-CoV-2, with suggestions that the glycosylation status of IgG3 affects the SARS-CoV-2 binding specificity to the S protein [46]. Here, we will compare the overall population antibody responses observed in population studies.

### 2.2. Pfizer/BioNTech BNT162b2 COVID-19 Vaccine Antibody Responses

Pfizer and Moderna are examples of mRNA-based vaccines. These act by injecting the RNA sequence coding for the modified antigen S protein to elicit an immune response [18]. The mRNA vaccines use lipid nanoparticle (LNP) technology developed initially by Canadian Acuitas Therapeutics Inc, and then Pfizer and BioNTech utilized this technology in development of the mRNA vaccines BNT162b1 and BNT162b2 which were later chosen as vaccines [47]. The mRNA vaccines encode the modified spike protein stabilized in a prefusion conformation, allowing the immune system to respond to the virus at the prefusion stage prior to SARS-CoV-2 cell entry. The mRNA-based vaccines were initially shown to be effective and underwent safety evaluation, progressing through clinical trials and being approved initially under emergency use agreement (EUA) licensing for use in several nations [25]. A phase 1/2 study initially evaluated RBD-binding IgG concentrations and SARS-CoV-2-neutralizing titers in sera (*n* = 45) [48]. IgG antibodies were found to increase with dose, and bindings to the RBD of the S1 protein were shown to be induced [49]. In phase 3 clinical trials, mRNA vaccines showed a 95% efficacy [50]. After approval in December 2020, Pfizer and BioNTech began distributing BNT162b2 throughout the world [51]. Subsequently, studies of real-world vaccine recipients began recruiting larger cohorts, characterizing in detail the immune responses elicited by BNT162b2 and memory B/T cells to the infection in relevant antibody sub-types (IgG, IgM, IgA), thus clearly outlining the effectiveness and protection of BNT162b2. SARS-CoV-2 transmitted between humans occurs mainly via respiratory routes. Therefore, it is important to investigate whether IgA is also developed after either infection or vaccination, due to its role in mucosal protection [52]. A study (*n* = 108) found that IgA against S protein (S1) and the receptor-binding domain (RBD) was produced in serum 1 month after one and two doses of BNT162b2, but this was not measurable as secretory IgA with serum IgG and IgA decreasing after 6 months [53]. Interestingly, the study found that anti-S IgM increased with time in 10% of participants. Consequently, other studies (*n* = 27) verified the comparison of serum IgA and IgG in naïve/infected vaccine recipients. Antibody and cytokine responses against S1, RBD, and whole-spike protein responses showed that SARS-CoV-2-specific B memory cells did induce an adaptive response-measured T cell-evoked cytokine production, defined by IL-2, IL-4, IL6, IL-10 and TNF-α responses, as well as chemokine (CCL2, CXCL10) production [54]. Other detailed studies (*n* = 12) followed BNT162b2 with a second dose, indicating that T cell expression, as measured by clusters of differentiation markers (CD4+/CD8+) in vaccine participants, also produced IFN-γ, which is indicative of humoral and cellular responses against SARS-CoV-2 epitopes [55]. Another study (*n* = 20) that specifically researched early immune responses after one dose of BNT162b2 tracked T cell and antibody responses. This study found that, at day 10, 50% (10/20), 85% (17/20), and 80% (16/20) of responses had a marked 4× increase, as measured by fluorescence in IgM, IgA and IgG. Interestingly, in this study, it was confirmed that 20% (4/20) of participants had measurable IgG anti-S that completely blocked the ACE2 receptor and, as measured by a virus neutralization assay, that only 15% (3/20) of participants had nAbs, suggesting that these nAbs may not be required for early protection. Therefore, other corresponding studies (*n* = 163) clarified that infection-naïve and naturally infected people displayed significantly higher levels of IgG responses 12 days after one dose of BNT162b2 [56]. A comprehensive study (*n* = 871) on participants who received two doses of BNT162b2 quantified total IgG anti-S protein responses (which were assessed in serum samples at different time points for 3 months) to clarify that IgG anti-S protein levels continuously increased without variations in the levels of anti-SARS-CoV-2 S responses between sexes. However, IgG peaked following the second dose of the BNT162b2 vaccine, but a significant decrease occurred at 3 months in elderly participants [57]. This decline at 3 months concurs with other similar studies [58]. Therefore, research was conducted to evaluate the IgG anti-S protein response 6 months after BNT162b2 and found that IgG, produced in response to BNT162b2, began to decline after the second month of vaccination [59]. IgG anti-S protein was quantified to show a peak IgG response occurring at 2 months and decreasing over the next 4 months, with an average 6.3% peak titer remaining at 6 months. These significant differences occurred in participants of all ages [60,61]. A concurrent study (*n* = 92) analyzed the IgG anti-S protein 7 months after the second dose of BNT162b2, finding that IgG titers were reduced by 92% in cohorts that had prior confirmed SARS-CoV-2 infection and those who did not. IgG titers remained detectable throughout the study periods [62]. Since BNT162b2 contains RNA sequence coding only for the spike protein of the SARS-CoV-2 virus, this raises the possibility that other proteins could be elicited by the BNT162b2 COVID-19 vaccination. Y. Yoshimura et al. thoroughly compared IgG anti-N and anti-S in people vaccinated with two doses of BNT162b2, finding that the IgG anti-S protein developed, while the anti-N protein IgG against N (nucleocapsid) protein did not [63]. This makes sense given that the mRNA-based COVID-19 vaccine codes specifically for the spike protein of the SARS-CoV-2 virus. In addition to anti-S and anti-N IgG, another study quantified individual SARS-CoV-2 protein antibody responses against anti-RBD, anti-S1 and anti-S2 proteins, finding that BNT162b2 induces the production of antibodies against all S antigens within two weeks, while the second dose causes peak levels of antibodies in all participants, except for anti-N antibodies which remained negative before and after vaccination [64]. Many studies report antibody correlation with an initial increase and decrease in IgG after a certain amount of time following one or two doses of BNT162b2 [65]. Therefore, a third dose of BNT162b2 is currently being implemented, which also demonstrated a 95.3% efficiency in preventing SARS-CoV-2 infections in phase 3 clinical trials [66]. Evidence shows that there is a decline in the probability of contracting SARS-CoV-2 after vaccination [67]. In a comprehensive study (*n* = 550,232), this evidence indicated a risk ratio of 0.1428 at 65 days, or an 86% risk reduction after the second or third dose. Therefore, recent research clarified that, before the first BNT162b2 dose was given, participants with prior infection already had IgG anti-S protein antibodies, and that after the first dose, this antibody response was higher than that of naïve participants who had not been infected. This finding was expected, with vaccine-induced immunity acting as a booster of naturally occurring immunity. Therefore, these results suggest the possibility that delaying the second vaccine dose for those who had a previous COVID-19 infection may be beneficial [54,56,68,69,70,71]. In a recent study (*n* = 62), other authors considered whether the IgG anti-S protein may be attenuated in response to a second BNT162b2 vaccine dose [72]. One dose of BNT162b2 is reported to be protective against SARS-CoV-2 reinfection in previously infected people [73]. BNT162b2 appears to induce a strong systemic humoral response quantified in serum samples, but low salivary IgG and even lower secretory IgA antibodies. Serum IgA levels appear to peak after a single injection of the vaccine and do not increase after a second vaccine dose [52]. Furthermore, 8 months after BNT162b2 vaccination, people with and without prior SARS-CoV-2 infection demonstrated similar levels of memory SARS-CoV-2-specific B cells [54,74]. Therefore, given the above research, it appears that serum anti-S protein IgG antibodies are immunologically active from 12 days, peaking after 2–3 months and declining at 6 months in sera with low levels of IgM (a marker of early infection) and similarly low levels of secretory IgA in either infection/vaccine responses. Mutations in S protein epitopes, including E484K and K417T/N, have resulted in increased transmissibility; therefore, cellular B cell responses were analyzed by measuring further B cell markers (CD21, CD27, CD71) to determine the activation of these CD27+ CD38+, CD71+ phenotypes [74]. D. Mileto et al. evaluated the anti-S IgG response one month after the second dose of a vaccine countering Alpha, Gamma, and Eta variants. They found, in neutralization assays, that anti-S IgG responses were protective against SARS-CoV-2, with epitopes present in the Beta and Delta strains that demonstrated partial immune evasion [75]. Another project utilizing transcriptomic analysis determined that one dose of BNT162b2 elicited a recall response of IgA plasmablasts targeting the S2 protein subunit, with IgG plasmablasts expanding to target the S1 RBD from the naive B cell pool [76]. This response was strongly boosted by the second dose and delivered nAbs against SARS-CoV-2, concurring that the original Wuhan-Hu-1 variant evoked a stronger immune response of IgG anti-S SARS-CoV-2. However, this immunological evasion of the SARS-CoV-2 variants could be prevented by two doses of BNT162b2, promoting the formation of memory B cells and RBD-specific antibodies to eliminate the SARS-CoV-2 variants [76]. Recent evidence showed that that Alpha and Delta variants were less able to evade nAbs, compared to the Beta and Omicron variants [77]. Therefore, B cell development, antibody production, and subsequent circulation can produce immunological responses by generating anti-S protein RBD antigen-specific B cells. Indeed, a key component of vaccine effectiveness is the human memory B cell compartment [78]. As discussed earlier, after BNT162b2, memory B cells are formed and produce antibodies equivalent to those that drive the initial response [55]. Memory B cells generate IgA, IgM, and IgG isotypes, which are shown to counteract the effects of natural serum decline [53,74]. Another longer follow-up study demonstrated that memory B cells remained, although the apparent antibody reduction six months after BNT162b2 did provide long-lasting immunity [78,79,80]. In this study, Ciabattini et al., (n = 145) compared class-switching and memory cell markers, as measured within B cell plasmablasts, to determine spike protein specificity and class-switching immune recall responses to spike antigens. They measured sub-sets of B cells (CD19+) to assess the expression of CD19+, CD24+, CD27+, and CD38+-specific SARS-CoV-2 B cells. This key finding demonstrated that these B cells did produce a mostly IgG1 and IgG3 response and absence of IgG2 and IgG4, and that this recalled-memory immune response occurred with significant increases in spike protein-specific B-cell producing IgG and stable IgA producing B cells, at up to 6 months—with IgG memory specific B cells measured at 66% but also IgM specific B cells at 100% in vitro [78].

### 2.3. AstraZeneca COVID-19 Vaccine Antibody Responses

The Oxford–AstraZeneca (AZ) vaccine was developed from the DNA vaccine of a chimpanzee replication-deficient adeno-viral vector, delivering an encoded S protein antigen to target SARS-CoV-2 infection [18,35]. This immunogen was known as ChAdOx1, and later as AZD1222, and was developed by the University of Oxford in collaboration with AstraZeneca. AZD1222 consists of a replication-deficient adenovirus vector that expresses full-length coronavirus spike protein and stimulates the development of B cells to produce antibodies and T cells [81]. In a cohort sample (*n* = 380), the IgG anti-S protein response against COVID-19 infection was sufficient, with peak IgG anti-S occurring at 107, 101.5, and 70.2 days, respectively, in age groups of 18–55, 56–69, and over 70 years. However, the AZD1222/BNT162b2 combination was also measured by IgG generated against S1, RBD, full-length spike proteins, and T cell responses, as measured by IFN-γ production [82,83]. In phase 1/2 clinical trials, it was demonstrated that IgG-specific SARS-CoV-2 antibodies, induced after AZD1222 vaccination, peaked at 14–28 days with no significant difference between immunogens [82,83,84,85]. Phase 2/3 trials of AZD1222 showed an overall 70.4% efficacy, and it was initially used under emergency use authorization (EUA) as before [84,85,86]. Subsequently, the AZD1222 vaccine was measured in real-world data, and its first doses were reported to produce equivalent levels of antibody and T cell responses. Therefore, COVID-19 hospitalization rates significantly decreased after one dose of the AZD1222 vaccine [87]. Clinical trial studies were then conducted in a real-world setting. Müller et al. reported that AZD1222 vaccination mostly evoked antibody isotypes of IgM and IgG [88]. Furthering this, an evaluation occurred of serum IgG and IgA responses against each of the SARS-CoV-2 subunit domains (S1, S2, RBD, and N proteins) of three SARS-CoV-2 variants (Alpha, Beta and Gamma) and found that IgG to each of these four SARS-CoV-2 proteins increased from 4 to 12 weeks, with antibodies against S2 having significantly lower IgA responses to each variant—which is line with other research studies before [89]. It was also found that the first AZD1222 vaccine dose enhanced serum IgA between 4 to 12 weeks [89]. Other studies found that, after infection, a single dose of AZD1222 was enough to protect SARS-CoV-2 patients from reinfection and functioned as a booster dose [90,91,92]. Following the first dose, elevated titers gradually decreased between 56 and 76 days later in those previously infected with COVID-19 [93]. According to research by Hoque et al., IgG anti-RBD/S1 protein responses, elicited after the first and second doses of the vaccine, were 99.9% and 100%, respectively, in naïve vaccine recipients, with no sex-specific differences, based on 74 days from initial dose [94]. Therefore, this extended interval between the two doses appeared to be better in vaccine recipients compared to the standard interval [93,95,96,97]. Investigation shows that, following the first dose, the AZD1222 vaccine produced a strong humoral reaction, which remained an indicator following the second vaccine dose. In this study, the sensitivity of different assays was compared after the first dose of AZD1222, showing a range of 75.4% to 89.3% seropositivity [98,99]. These responses were not correlated with age or gender but showed a dramatic reduction in nAbs from 70.1% (4 weeks) to 49.2% (8 weeks) [100]. Following the second AZD1222 dose, IgG anti-S protein levels peaked after 21 days and gradually decreased after that. IgG antibodies were consistent in serum for up to six months [101,102,103,104,105]. Likewise, nAbs induced by AZD1222 immunization began to decline [106,107]. Other evidential studies report that, after the first AZD1222 dose, a single dose of the AZD1222 vaccine did not result in a significant antibody decline at up to 11 weeks [108]. The absence of N protein in AZD1222 also resulted in no detectable anti-N IgG antibodies in serum [109]. IgG anti-RBD antibodies from people vaccinated with AZD1222 were broadly cross-reactive against multiple VOCs and had neutralization potency against the Beta and Delta variants [110]. Another report showed that this response was still observed one month after the first AZD1222 vaccine dose but did not prevent mild or moderate COVID-19 infection by the Beta variant [111]. In contrast, AZD1222 vaccination prevented SARS-CoV-2 infection by the Alpha and Delta variants [112,113]. Despite the Omicron variant’s ability to avoid nAbs, AZD1222 (*n* = 3513) was shown to lower the chance of acquiring pneumonia, in individuals who received two vaccine doses, for up to 6 months [114]. Furthermore, the transmission of the Alpha and Delta variants was reduced after receiving the AZD1222 vaccine [115].

### 2.4. Sinopharm COVID-19 Vaccine Antibody Responses

The third COVID-19 vaccine to be compared in this paper is Sinopharm (BBIBP-CorV), an inactivated virus combined with an alum adjuvant made from virus particles and grown in culture and thus improving immunogenicity [18]. BBIBP-CorV was created by the Beijing Institute of Biological Products and has a 79% efficacy [116]. Since BBIBP-CorV uses a completely inactivated virus, it is predicted to trigger an immune response against all SARS-CoV-2 proteins: nucleocapsid (N), membranes (M), envelope (E) proteins, and spike proteins (S) [117]. Along similar lines, BBIBP-CorV underwent licensing agreements with the WHO updated guidelines for usage updated in June 2022. A significant amount of nAbs were produced by BBIBP-CorV in preclinical studies, with 100% seroconversion occurring at 21 days [118]. In phase 1/2, it was shown that healthy individuals tolerate and respond well to BBIBP-CorV 4 days after the initial vaccine dose, and substantial virus-specific antibody responses were detected [119,120]. On day 42, it was found that a lower vaccine dose had a higher level of nAb response [120]. After approval, a real-world study showed a lower seroconversion rate than in phase 1/2; despite this decline in seroconversion, BBIBP-CorV was protective against chronic SARS-CoV-2 infection [121,122]. A similar finding was also reported in the under-18 age group [119]. Research shows that the administration of BBIBP-CorV stimulates IgG response, lowers mortality, and prevents infection [123]. BBIBP-CorV was shown to induce nAbs and IgG against RBD after two vaccine doses; compared to males, females had greater responses [124]. A notable difference between the nAbs titers in different age ranges was their tendency to be found at lower levels in older age ranges. IgG and IgM spike-specific antibody concentrations were comparable to nAbs levels, which peaked at 14 days following the second dose, after being at a low level following the first dose [124,125]. When comparing vaccine-induced immunity between participants with prior SARS-CoV-2 (*n* = 126) and naïve infection, it was found that IgG anti-RBD and nAbs were detected in 87.06% and 78.82% of participants after two doses, with T cells waning after 1 dose specific to S, M and N proteins [124]. In this study (*n* = 126), Li et al., measured T cell cytokine production by IFN-γ, IL-2, and TNF-α by the restimulation of T cells with peptides from S, M, and N proteins antigens. They concluded that the T cell response occurred in T helper cells (CD4+) and cytotoxic T cells (CD8+) at a ratio of 95.83%:54.16% within a cohort of double-vaccinated participants to be sustained [124]. Additionally, a key finding of this study was that T cell response was low in comparable inactivated vaccines after one dose but was demonstrably sustained after two doses [124,126,127,128,129]. Despite the overall and consistent waning of antibody responses, memory B cell responses were still maintained and provided protection [130,131]. Compared to the original SARS-CoV-2 strain, the BBIBP-CorV vaccine was less efficient in neutralizing other variants, which was consistent with prior studies [132,133]. Research reported that an additional third BBIBP-CorV vaccine dose significantly increased antibody responses compared to a two-dose vaccine schedule and compensated for antibody decline [134]. Furthermore, booster doses were shown to induce nAbs against other SARS-CoV-2 variants, with these variations observed between Alpha and Beta variants, with the Beta variant displaying more resistance to IgG-evoked responses than two vaccine doses [133,135]. After BBIBP-CorV vaccination, antibody levels were found to be affected by both gender and age, with females and young people having higher antibody responses [128,136,137].

### 2.5. Novavax NVX-CoV2373 COVID-19 Vaccine Antibody Responses

The initial approval process of Novavax Nuvaxovid (NVX-CoV2373), later known as Covovax, also began in 2020 along similar lines; this protein subunit vaccine platform employed by Novavax represents a traditional vaccine technology trusted for many decades, with the first production of a recombinant subunit vaccine occurring against Hepatitis B in the mid-1980s [138]. Similarly, approval occurred under emergency use listing in December 2021. NVX-CoV2373 consists of recombinant baculovirus production and stabilized SARS-CoV-2 spike protein from the ancestral strain. This protein is embedded in a buffered polysorbate 80 micelle and is adjuvanted with Matrix-M, which is saponin based [139]. Clinical evaluation of NVX-CoV2373 found it to be safe and immunogenic in adults [140]. It showed a high vaccine efficacy against severe COVID-19 (>90%) in a study comprising 29949 participants exposed to multiple variants of concerns (VOC) circulating in the United States and Mexico at that time [141]. This clinical trial resulted in the determination that vaccine efficacy was 90.4% (95% CI, 82.9 to 94.6; *p* < 0.001). Concurrently, in an earlier phase 1–2 trial, it was found that NVX-CoV2373 vaccine elicited a CD4+ T-cell response, with an immune response directed towards a T_H_1 phenotype. Antibody titers were measured at different time points and for different dosage regimens as before. It was demonstrated that a two-dose 5-μg regimen was optimal for antibody production, and analysis at day 35 revealed that antibody titers for anti-spike IgG antibodies and neutralization responses exceeded those seen in convalescent serum from COVID-19 patients (63,160 vs. 8344 and 3906 vs. 983 ELISA units, respectively) [140]. A recent study also evaluated the induction of antibodies capable of neutralizing Omicron sub-lineages (including BA.2, BA.4, BA.5, BA.2.12.1) following two or three doses of NVX-CoV2373. The authors report that after two doses, Omicron sub-lineages BA.1 and BA.4/BA.5 were resistant to neutralization in 72% (21/29) and 59% (17/29) of the samples. A third dose of NVX-CoV2373 vaccine, however, showed high titers against Omicron BA.1 (GMT: 1197) and BA.4/BA.5 (GMT: 582), with titers comparable to those triggered by three doses of an mRNA vaccine. Due to the dominance of BA.4/BA.5 in multiple locations, these results are of great importance and highlight the potential value of the NVX-CoV2373 vaccine as a booster in resource-limited environments [142]. A head-to-head comparison between BioNTech/Pfizer and Novavax revealed that two doses of NVX-CoV2373 strongly induced anti-spike IgG, although IgG-levels were lower than after vaccination with a dual dose of BNT162b2 or mRNA-1273 (*p* = 0.006). Irrespective of which vaccine was used, and regardless of different IgG-levels, neutralizing activity towards VOCs was found to be highest for Delta, and next was BA.2 followed by BA.1. In the NVX-CoV2373 subunit vaccine, an induction of any spike-specific CD8+ T-cells was only detectable in 3/22 (14%) samples. Contrastingly, induction of spike specific CD4+ T-cells was detected and present in 18/22 (82%) individuals, although to a lower overall median level (*p* < 0.001). Also, the expression levels of CTLA-4 were lower (*p* < 0.0001), with less multifunctional cells co-expressing IFN-γ, TNF-α and IL-2 (*p* = 0.0007) reported. In addition to neutralizing antibodies, NVX-CoV2373-induced CD4+ but also CD8+ T-cells similarly recognized all tested VOCs from Alpha to Omicron. Furthermore, for individuals with prior SARS-CoV-2 infection, one dose of NVX-CoV2373 showed immunogenicity to a similar level compared to two doses in non-infected individuals [143].

### 2.6. Pfizer vs. AstraZeneca vs. Sinopharm Vaccines Antibody Responses

In comparison to natural SARS-CoV-2 infection-induced immune responses, antibody responses induced by BNT162b2 appear higher, whereas those induced by BBIBP-CorV appeared to be lower, after one dose [144,145]. Therefore, other population studies and serology studies have detailed the length and breadth of IgG anti-S protein response with accompanying statistical analyses (*n* = 7256). These estimations and models suggest that 24% of participants may not develop anti-S protein antibodies, and they predict that anti-S protein IgG can protect against reinfection and has a half-life between 184 days and 1.5–2 years [146]. Whilst other population studies (*n* = 212,102) compare standard laboratory-based assays (ELISA) with proprietary lateral flow immunoassays (LFIAs)—the comparison of the accuracy of these self-tests (*n* = 5000) showing sensitivity and specificity ratios of 82.1%/97.8%, respectively—the measuring of S1 protein antigens will undoubtedly be useful in the future and requires further research [146]. Therefore, having considered the overall profiles (above) of B cell-produced IgG antibodies in SARS-CoV 2 infections and vaccine immune responses, it will be necessary to clarify the roles of many other cell sub-types in our following article awaiting peer review [147]; these will also include B cell subsets, and T cells (according to current classifications) responsible for variations in infection or vaccination immune responses.

## 3. Limitations

All assays utilized in the above studies—RT-PCR, ELISA, LFIA, CLIA, etc.—are subject to validation for accuracy, specificity, and sensitivity. The current quantification of the correlation of protection through antibody responses is a guideline. The available quantification of antibody serology varies and is subject to standardization or international standards reported as ng/mL, IU/mL (nAbs), or BAU/mL for binding assays [148,149,150,151]. SARS-CoV-2 variants have different epitope profiles through mutational evolution. Current real-world epitope analysis of SARS-CoV-2 variants in infection or after vaccination will undoubtedly lead to understanding how epitopes in different pathogens, as well as SARS-CoV-2, affect overall innate and adaptive immune response (as measured by B and T cell subsets and cytokine production). However, further research is needed to understand why different pathogens evoke such differential immune responses (healthy adult participants and two-dose vaccine cohorts were included in this analysis) [152,153,154]. It is of note that other studies investigating vaccine hesitancy (*n* = 460) have shown that rates of hesitancy in vaccine uptake were estimated at 42.2% for COVID-19 vaccines in comparison to 10.9% of other childhood vaccines; this would be interesting to compare in other research studies in the future [155]. Also, there are now many other vaccine platforms in development, including dendritic cell vaccines, CAR-T (chimeric antigen receptors-T) cell vaccines, and others not discussed here, that may have far reaching therapeutic uses in other pathological therapeutic treatments in the coming years [156]. Further information is available on supplementary materials as above below on all cited articles or statistical analysis cited. 

## 4. Conclusions

The correlations of innate and adaptive immunity regarding current humoral and cellular immune response studies have been detailed to examine the immunological profile of either natural infection or vaccine-induced serological responses against SARS-CoV-2 immunogens across populations. A specific SARS-CoV-2 and vaccine-induced antibody response occurs that is specific to pathogen epitopes that elicit immunogenicity. However, further research is required to elucidate the T cell response in infection and/or vaccine responses to SARS-CoV-2, which displays higher infection rates than other comparable HCoV pathogens.

## Data Availability

All related data is presented in this paper. Additional inquiries should be addressed to the corresponding authors attached above or below supplemental data sheets. Brent Brown (B.B.); ORCiD (0000-0001-5238-6943), Ingo Fricke ORCiD (0000-0001-7638-3181). Chinua Imarogbe ORCiD (0000-0002-8200-0885); Suhaila A. Al-Sheboul ORCiD (0000-00019001-3232).

## Supplementary Materials

The following supporting information can be downloaded at: www.covidvaccineresearch.org/publications or https://www.ndm.ox.ac.uk/COVID-19/COVID-19-infection-survey/results/longer-articles, COVID-19 Vaccines Advice (who.int) The Pfizer BioNTech (BNT162b2) COVID-19 vaccine: What you need to know (who.int); The Oxford/AstraZeneca (ChAdOx1-S [recombinant] vaccine); The Sinopharm COVID-19 vaccine: What you need to know (who.int), The Novavax vaccine against COVID-19: What you need to know (who.int).
